# Supplementary material for: Prevalence and Characteristics of Metabolic Hyperferritinemia in a Population-Based Central-European Cohort
Source: Biomedicines. 2024 Jan 17;12(1):207. doi: 10.3390/biomedicines12010207 (PMC10813305; doi:10.3390/biomedicines12010207)
Supplement: Supplementary file 1 [file biomedicines-12-00207-s001.zip › biomedicines-2796322-supplementary.pdf]

Supplementary material

Table S1: Main characteristics in total numbers, percentages (%) or median and interquartile range (IQR) for the affirmation cohort. Abbreviations: HF hyperferritinemia, BMI body mass index, Waist waist circumference, ALT alanine transaminase, AST aspartate transaminase, Gamma GT Gamma-glutamyltransferase, ALP alkaline phosphatase, HDL high-density lipoprotein, LDL low-density lipoprotein, TG triglycerides, Hb blood hemoglobin, WBC white blood cells, PLT platelets. The asterisk (*) denotes significant difference between the non-HF and the HF group at $p < 0.05$

	**Non-HF**	**HF all grades (n = 857)**		
	**n = 5567**	**HF 1 (n = 774)**	**HF 2 (n = 79)**	**HF 3 (n = 4)**
**Age 40-49 years**	26% (n = 1461)	All grades 17% (n = 145)		
		17% (n = 133)	15% (n = 12)	0% (n = 0)
**Age 50 – 59 years**	45% (n = 2501)	All grades 44% (n = 375)		
		43% (n = 334)	48% (n = 38)	75% (n = 3)
**Age 60-69 years**	26% (n = 1438)	All grades 36% (n = 307)		
		36% (n = 280)	33% (n = 26)	25% (n = 1)
**Age ≥ 70 years**	3% (n = 159)	All grades 4% (n = 30)		
		3% (n = 27)	4% (n = 3)	0% (n = 0)
**Male***	45% (n = 481)	All grades 69% (n = 590)		
		66% (n = 510)	96% (n = 76)	100% (n = 4)
**Female***	55% (n = 3086)	All grades 31% (n = 267)		
		34% (n = 264)	4% (n = 3)	0% (n = 0)
**BMI kg/m2***	26 (23-29)	28 (25-31)	29 (26-32)	30 (28-31)
**Alcohol g/d***	6 (2-15)	11 (4-21)	13 (4-27)	31 (15-38)
**ALT U/l***	21 (16-28)	29 (22-39)	38 (28-60)	86 (52-164)
**AST U/l***	22 (19-26)	25 (21-31)	30 (25-42)	65 (38-112)
**GammaGT U/l***	21 (15-33)	32 (22-50)	41 (27-62)	126 (86-384)
**ALP U/l***	63 (53-75)	65 (55-77)	64 (50-76)	76 (64-92)
**HDL mg/dl***	62 (51-75)	54 (45-66)	46 (40-52)	58 (43-68)
**LDL mg/dl***	139 (116-164)	148 (125-170)	138 (114-172)	144 (118-179)
**TG mg/dl***	94 (69-131)	120 (90-170)	165 (125-229)	216 (134-284)
**Hgb g/dl***	14.2 (13.5-15.1)	14.9 (14.1-15.6)	15.2 (14.7-16.0)	15.8 (14.8-16.7)
**WBC 10^9/L***	5.8 (4.9-6.8)	6.1 (5.1-7.2)	5.7 (5.1-6.8)	5.6 (5.2-7.0)
**PLT 10^9/L***	248 (214-283)	239 (207-273)	208 (172-234)	212 (192-236)

Table S2: Presence of fulfilled metabolic criteria within the HF and non-HF groups in percentages and numbers for the affirmation cohort. Abbreviations: HF hyperferritinemia. The p-value indicates significant difference between the non-HF and the HF group at $p < 0.05$

	**Affirmation cohort n = 6424**	**HF all grades n = 857**			**Non-HF n = 5567**	**p-value**
		**HF 1 n = 774**	**HF 2 n = 79**	**HF 3 n = 4**		
**Fulfilled metabolic criteria**	60% n = 3878	All grades 84% (n = 719)			57% n = 3159	**< 0.001**
		83% n = 641	94% n = 74	100% n = 4		

Table S3: Presence of major or minor metabolic characteristics within the different groups in percentages and numbers for the affirmation cohort. Numbers for the minor criteria are counted in the remaining subjects after application of the major criteria. Abbreviations: HF hyperferritinemia. The p-value indicates significant difference between the non-HF and the HF group at $p < 0.05$

**MAJOR CRITERIA WITHIN THE AFFIRMATION COHORT**						
	**Study cohort n = 6424**	**HF all grades n = 857**			**Non-HF n = 5567**	p-value
		**HF 1 n = 774**	**HF 2 n = 79**	**HF 3 n = 4**		
Presence of a major criterion	36% n = 2282	All grades 63% (n = 540)			31% n = 1742	< 0.001
		61% n = 472	81% n = 64	100% n = 4		
**MINOR CRITERIA WITHIN THE REMAINING COHORT**						
	**Remaining cohort n = 4142**	**Remaining HF all grades n = 317**			**Remaining non Hf N = 3825**	p-value
		**HF 1 n = 302**	**HF 2 n = 15**	**HF 3 n = 0**		
Presence of ≥ 2 minor criteria	39% n = 1596	All grades 56% (n = 179 of 317)			37% n = 1417	< 0.001
		53% n = 169	3% n = 10	0% n = 0		

Supplementary Table S4: subgroup analysis of the general characteristics for the Non-HF-group and the HF 1 group, numbers represent median and interquartile range (IQR).

	Non-HF (N=8,527)	HF 1 (N=1,225)	p-value
ages	55 (49-61)	58 (52-64)	<0.001
BMI kg/m2	25 (23-29)	28 (25-31)	<0.001
Waist cm	92 (83-100)	99 (92-107)	<0.001
Alcohol g/d	7 (2-17)	13 (4-27)	<0.001
ALT U/l	21 (16-28)	28 (21-39)	<0.001
AST U/l	22 (19-27)	25 (21-31)	<0.001
GammaGT U/l	21 (15-32)	32 (22-51)	<0.001
ALP U/l	63 (53-75)	65 (56-77)	<0.001
HDL mg/l	63 (52-75)	55 (46-68)	<0.001
LDL mg/l	139 (115-164)	148 (124-171)	<0.001
TG mg/dl	94 (69-132)	118 (89-167)	<0.001
Hgb g/dl	14.2 (13.4-15.0)	14.9 (14.1-15.6)	<0.001
WBC 10^9/L	5.8 (4.9-6.9)	6.1 (5.1-7.2)	<0.001
PLT 10^9/L	247 (213-283)	237 (205-271)	<0.001

Supplementary Table S5: subgroup analysis of the general characteristics for the Non-HF-group and the HF groups 2 and 3 combined, numbers represent median and interquartile range (IQR).

	Non-HF (N=8,527)	HF 1+2 (N=163)	p-value
Ages	55 (49-61)	58 (52-64)	<0.001
BMI kg/m2	25 (23-29)	29 (26-32)	<0.001
Waist cm	92 (83-100)	102 (96-112)	<0.001
Alcohol g/d	7 (2-17)	17 (6-34)	<0.001
ALT U/l	21 (16-28)	38 (28-58)	<0.001
AST U/l	22 (19-27)	30 (24-42)	<0.001
GammaGT U/l	21 (15-32)	48 (30-81)	<0.001
ALP U/l	63 (53-75)	64 (52-79)	0.45
HDL mg/l	63 (52-75)	49 (42-57)	<0.001
LDL mg/l	139 (115-164)	142 (117-173)	0.095
TG mg/dl	94 (69-132)	163 (118-219)	<0.001
Hgb g/dl	14.2 (13.4-15.0)	15.2 (14.7-16.1)	<0.001
WBC 10^9/L	5.8 (4.9-6.9)	6.0 (5.3-7.1)	0.005
PLT 10^9/L	247 (213-283)	209 (179-241)	<0.001
